# Potent human antibodies against SpA5 identified by high-throughput single-cell sequencing of phase I clinical volunteers’ B cells

**DOI:** 10.1016/j.isci.2024.111627

**Published:** 2024-12-18

**Authors:** Wenhao Wang, Xin Li, Yangxue Ou, Jinrui Zhou, Yaru Gu, Bixia Liu, Yan Zheng, Ying Wang, Rui Zhang, Quanming Zou, Qianfei Zuo, Bin Wang

**Affiliations:** 1 Institute of Respiratory Diseases, Xinqiao Hospital, Third Military Medical University (Army Medical University), Chongqing 400037, China; 2 Department of Microbiology and Biochemical Pharmacy, College of Pharmacy, Army Medical University, Chongqing 400038, P.R. China; 3 School of Pharmacy, Henan University, Kaifeng 475004, China; 4 Department of Tropical Medicine, College of Military Preventive Medicine, Army Medical University, Chongqing 400038, China; 5 Department of Cosmetology and Dermatology, Chongqing Hospital of Traditional Chinese Medicine, Chongqing 400021, China; 6 College of Medicine, Southwest Jiaotong University, Chengdu 610083, P.R. China; 7 College of Pharmacy and Bioengineering, Chongqing University of Technology, Chongqing 400038, P.R. China; 8 Department of Gastroenterology, Chongqing Key Laboratory of Digestive Malignancies, Daping Hospital, Army Medical University (Third Military Medical University), Chongqing 400042, P.R. China; 9 953th Hospital, Shigatse Branch, Xinqiao Hospital, Army Medical University, 857000 Shigatse, China; 10 Department of Clinical Laboratory, Chengdu Military General Hospital, Chengdu 610000, China

**Keywords:** health sciences, medicine, medical specialty, immunology

## Abstract

The drug resistance problem of *Staphylococcus aureus* needs to be solved urgently. Here, we report the rapid identification of *S. aureus* human antibodies by high-throughput single-cell RNA and VDJ sequencing of memory B cells derived from 64 volunteers immunized with recombinant five-component *S. aureus* vaccine (clinical phase I). From 676 antigen-binding IgG1^+^ clonotypes, TOP10 sequences were selected for expression and characterization, with the most potent one, Abs-9, having nanomolar affinity for the pentameric form of the specific antigen *S. aureus* protein A. Abs-9 also demonstrated strong prophylactic efficacy in mice injected with lethal doses of a wide range of drug-resistant *S. aureus* strains. Additionally, the potential epitopes were predicted and validated based on Alphafold2 and molecular docking methods. In all, this study screened for a potent strain of antibody that prevents infection with antibiotic-resistant *S. aureus*, providing important data to guide the design of vaccines based on antibody architecture.

## Highlights

• Six hundred seventy-six IgG1^+^ antigen-binding clonotypes were identified by high-throughput scRNA/VDJ-seq
• The antibody Abs-9 against SpA5 protects against lethal doses of *Staphylococcus aureus*
• Molecular docking predicted antigenic epitopes that bind to the antibody Abs-9

## Graphical abstract

**Figure:**
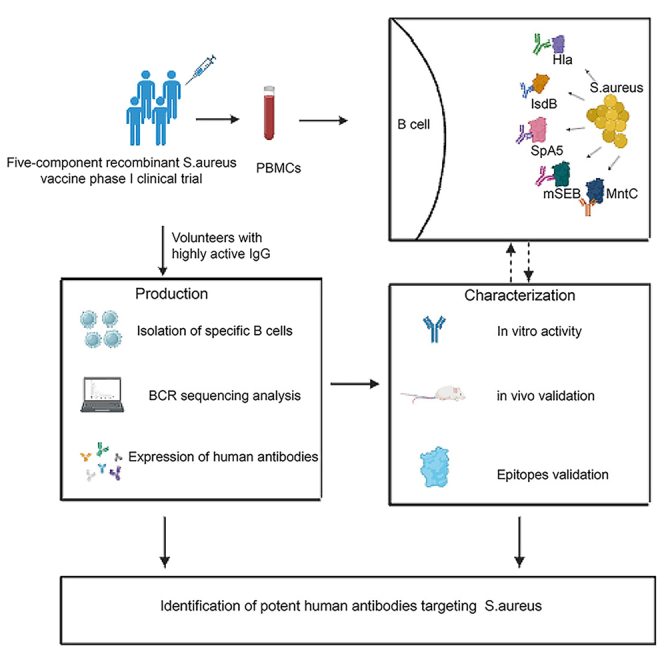

## Introduction

*Staphylococcus aureus* (*S. aureus*) is the major pathogen responsible for community-acquired and hospital-acquired infections.^1^^,^^2^^,^^3^ The abuse of antibiotics has produced drug-resistant strains, especially methicillin-resistant *S. aureus* (MRSA), which poses a serious threat to the safety of human life.^2^^,^^4^^,^^5^ Thus, prevention and treatment of *S. aureus* infections cannot be delayed.

The virulence factors produced by *S. aureus* infections play a crucial role in the pathogenesis, not only allowing successful immune escape of *S. aureus* but also interfering with the development of antigen-specific responses.^6^^,^^7^^,^^8^ Antibodies are one of the most important anti-virulence strategies to neutralize toxins and can provide prompt treatment for infected patients, making them valuable for in-depth studies of their preparation.^9^ Antibodies can neutralize bacterial virulence factors, blocking key mechanisms that bacteria use to evade the host immune system. Given their critical role in neutralizing pathogens, the development of effective antibodies has become a major focus in treating infections. Although *S. aureus* primarily causes skin infections, it can also lead to severe bloodstream infections such as sepsis. This progression may be influenced by protective immune memory mechanisms, suggesting that memory B cells could play a role in developing antibodies against *S. aureus* virulence factors.^10^

Currently, several methods have been developed to characterize antigen-specific B cells in human infected and vaccinated samples, but these methods are complex to implement and have low screening efficiencies.^11^^,^^12^^,^^13^ B cell receptor (BCR) sequencing is an effective method for responding to the level of immune response to infections and vaccinations, and the construction of antibody libraries by using BCR sequencing has the potential to screen for specific B cells, and the high-throughput individual memory B cell RNA and VDJ sequencing can be achieved to rapidly and efficiently identify neutralizing antibodies with therapeutic and prophylactic effects.^14^ Based on the aforementioned ideas, the development of fully human monoclonal antibodies by single specific memory B cells may become a new idea for the treatment of *S. aureus* infections.

The Engineering Center has independently developed a recombinant *S. aureus* vaccine containing five dominant antigens, namely, alpha-hemolysin (Hla), iron surface determining protein B (IsdB), mutant staphylococcal protein A (SpA5), mutant staphylococcal enterotoxin B (mSEB), and manganese ion binding protein C (MntC), as a result of its previous research on the immunological prevention and control of *S. aureus*.^15^^,^^16^ This vaccine is the only recombinant five-component *S. aureus* vaccine (rFSAV, relevant sequences are placed in the Data S1) undergoing phase III clinical trials in the world (CTR20221329). Here, we report the rapid identification of *S. aureus* human antibodies by high-throughput single-cell RNA and VDJ sequencing of memory B cells from 64 recombinant five-component *S. aureus* vaccine clinical phase I volunteers. From 676 antigen-binding IgG1^+^ clonotypes, TOP10 sequences were selected for expression and characterization, with the most potent one, Abs-9, having nanomolar affinity (1.959 × 10^−9^ M) for the pentameric form of the specific antigen *S. aureus* protein A (SpA5). Abs-9 also displayed strong prophylactic efficacy in mice infected with *S. aureus*.

Additionally, the 3D complex structure of mock antigen and antibody was modeled using computer-guided molecular docking method and showed the potential antigenic epitopes bound to the antibody Abs-9. In all, this study can guide vaccine design by screening human antibodies and predicting antigenic epitopes through antigen-antibody complex structure simulation prediction.

## Results

### Identification of specific memory B cells and antibody sequencing

**Figure 1:**
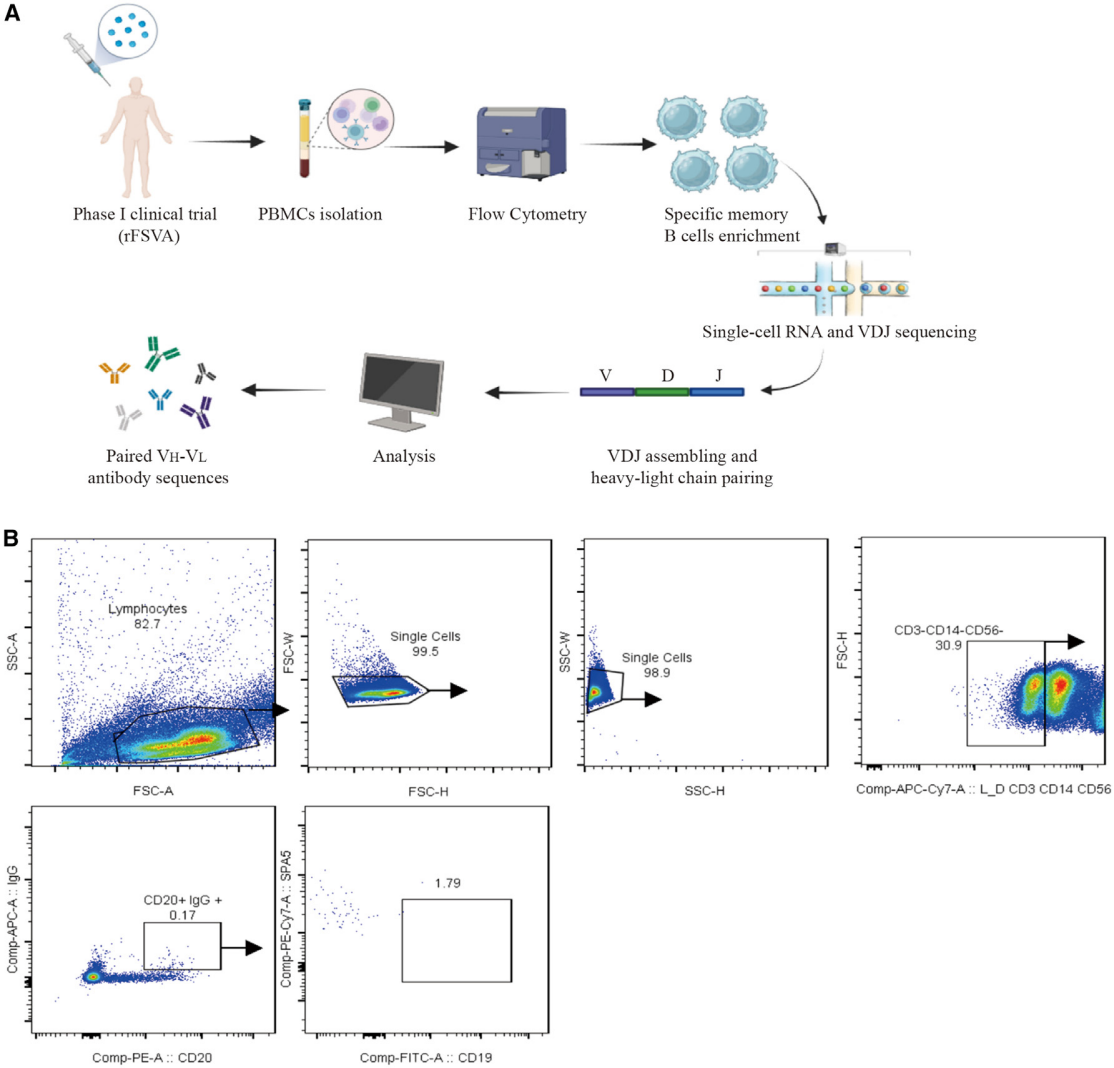
Acquisition of single specific memory B cells and results of BCR sequencing analysis (A) Flowchart illustrating the process of isolating single specific memory B cells via flow sorting for subsequent single-cell sequencing. (B) Flowchart depicting the procedure of sorting single specific memory B cells using flow cytometry.

**Figure 2:**
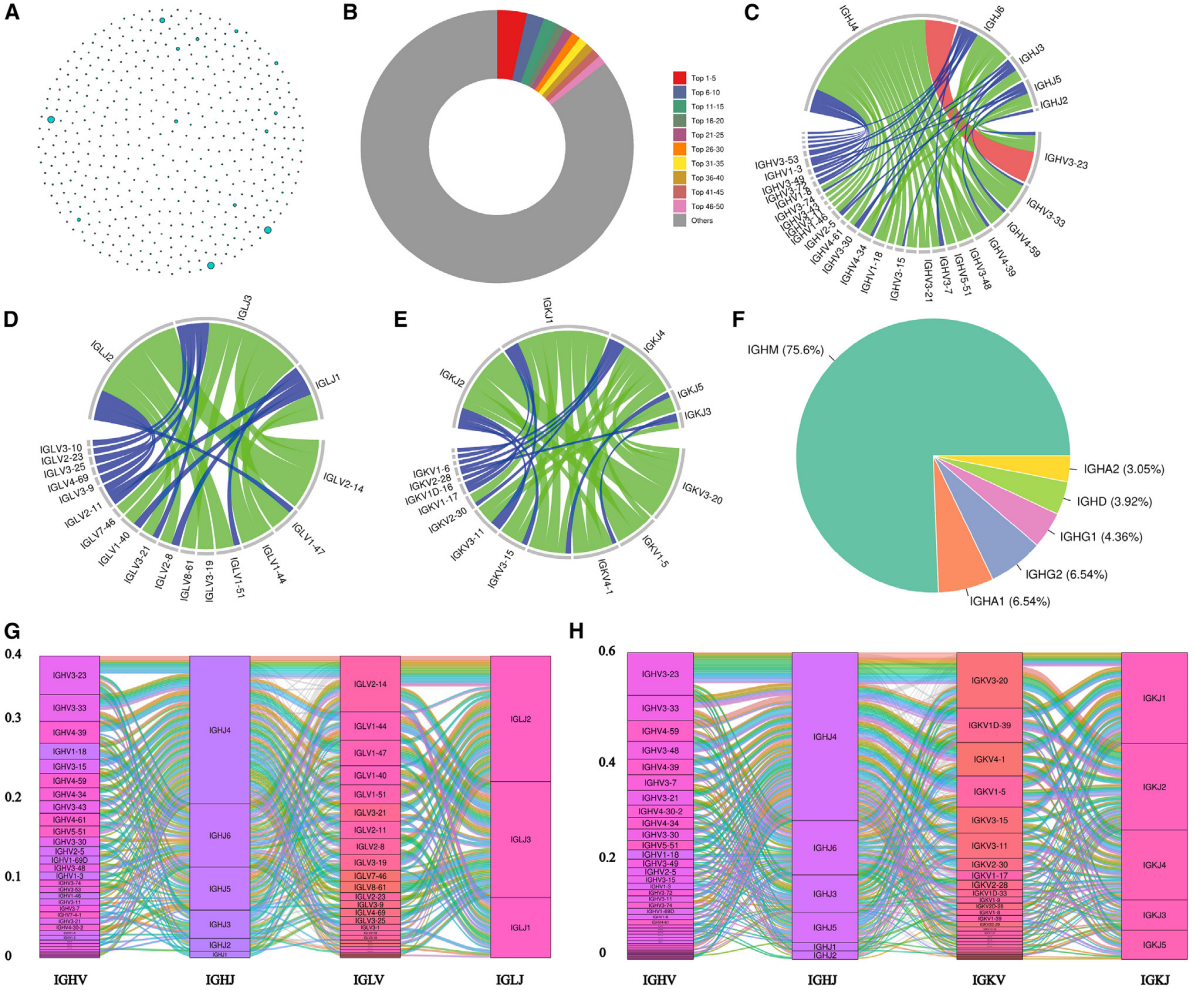
The results of BCR sequencing analysis (A) Clonotype network distribution graph representing the samples, where each node corresponds to a clonotype and node size reflects the abundance of the clonotype. (B) Ring diagram illustrating the percentage distribution of different clonotypes in the sample. (C) Chordal graph depicting the VJ gene usage for the top 100 abundant IgH chain clonotypes. The width of the connecting line between V and J indicates the frequency of VJ pair usage. Red indicates frequency >0.05, green indicates frequency between 0.01 and 0.05, and blue indicates frequency <0.01. (D) Abundance of the top 100 Igλ chain VJ genes presented using chordal plots. (E) Abundance of the top 100 Igκ chain VJ genes illustrated using chordal plots. (F) Plot displaying the proportion of cells expressing different BCR isoforms. (G) IgH-Igλ chain pairing abundance of top10 VJ pairs using shock plots. (H) IgH-Igκ chain pairing abundance of top10 VJ pairs using shock plots. (For interpretation of the references to color in this figure legend, the reader is referred to the web version of this article.).

To screen for specific memory B cells that bind to five *S. aureus* antigens, we co-incubated peripheral blood lymphocytes from phase I clinical subjects with biotin-labeled recombinant antigenic proteins, sorted them by flow cytometry (Figure 1B, relevant sequences are placed in the Data S1), and then performed high-throughput single-cell RNA and VDJ sequencing on quality-assured samples (Figure 1A). Bioinformatics analyses identified 10 pairs of highly expressed clonal immunoglobulin G (IgG) antibody variable and linker-region-expressing genes, including heavy and light chains (Figure 2). In conclusion, we screened IgG antibody sequences highly clonally expressed against five antigens from PBMCs of phase I clinical volunteers by high-throughput single-cell sequencing.

### The human antibody Abs-9 has a significant affinity for SpA5

**Figure 3:**
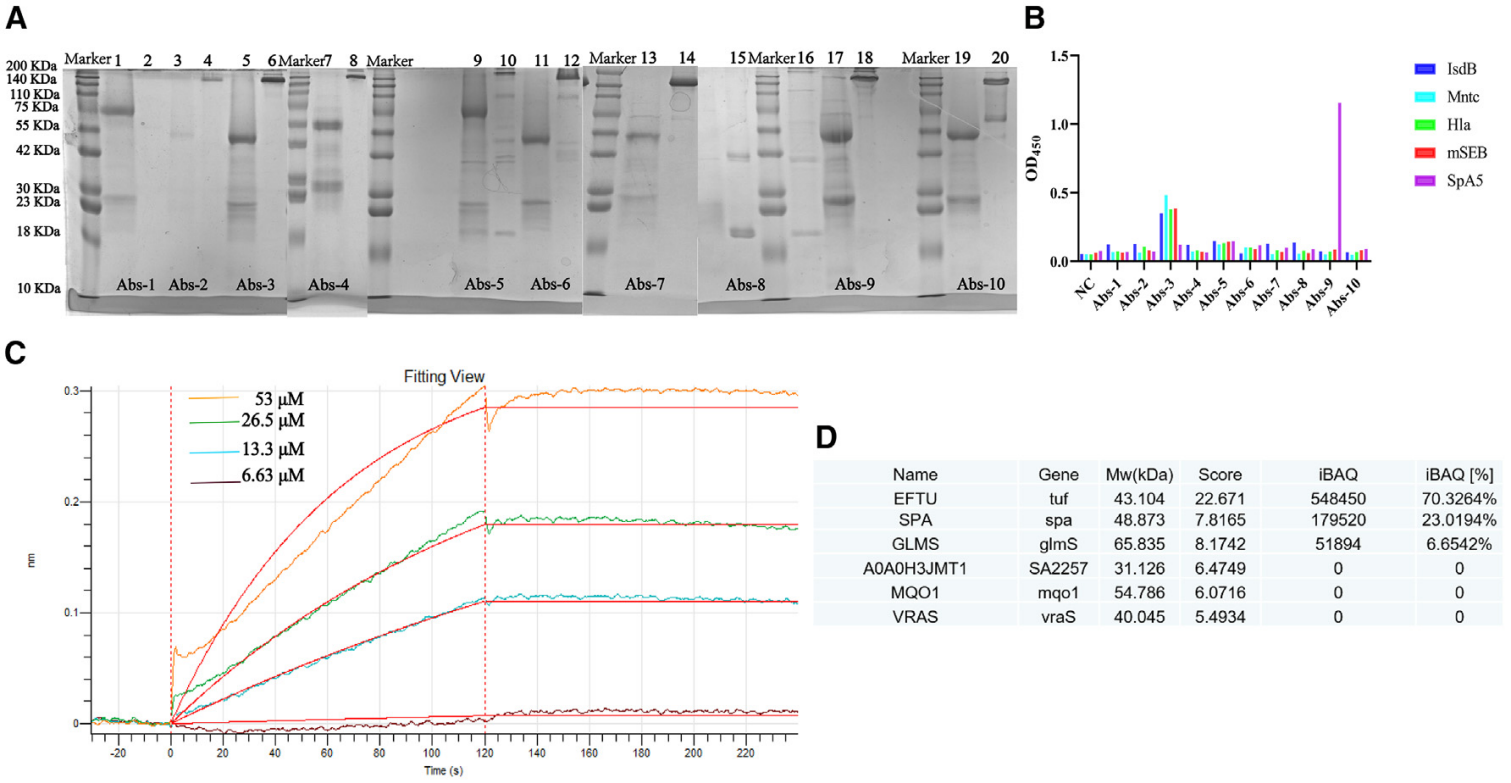
Characterization and binding activity of purified antibodies (A) SDS-PAGE gel displaying the purified top 10 IgG antibodies (Abs-1 to Abs-10), proteins were prepared by reducing and non-reducing conditions. (B) Assessment of the binding activity of top 10 antibodies toward five antigens at a dilution of 2 μg/mL. (C) BLI binding analysis of human antibody Abs-9 immobilized on a Pro A biosensor with different concentrations (6.63 μΜ–53 μΜ) of antigen SpA5. (D) Mass spectrometry of ultrasonically fragmented MRSA252 bacterial fluids co-incubated with antibody Abs-9 and collected by and eluted from protein A beads.

In order to screen out the antibodies with affinity for the five antigens, we constructed the heavy and light chain sequences of TOP10 antibody into a plasmid expression vector, transfected its amplified extracts, purified it, and identified it (Figure 3A). Enzyme-linked immunosorbent assay (ELISA) was used to detect the activity of antibodies against five antigens. The results showed that Abs-9 exhibited clear affinity for SpA5 (Figure 3B; relevant sequences are placed in the Datas S1 and S2). Based on the aforementioned experimental results, we started to characterize the affinity of antibody Abs-9 and used Biolayer Interferometry to measure the affinity of different concentrations of antigen SpA5 with antibody Abs-9, and after fitting the curve, we measured a KD value of 1.959 × 10^−9^ M (Kon = 2.873 × 10^−2^ M^-1^, Koff = 5.628 × 10^−7^ s^−1^), with a nanomolar affinity (Figure 3C).

In order to exclude the effect of non-specific binding of antigen SpA5, we ultrasonically fragmented and centrifuged the bacterial fluid of MRSA252, took the supernatant and coincubated it with antibody Abs-9 overnight, then bound it with protein A beads the next day, and collected the eluate for mass spectrometry detection. The experimental results showed that the antigen SpA5 is the specific antigen targeted by antibody Abs-9 (Figure 3D).

In conclusion, we successfully expressed and identified 10 pairs of highly expressed cloned IgG antibody sequences. Given the role of SpA5 in *S. aureus* immune evasion, we focused on screening antibodies with high affinity for this antigen. Our results show that the human antibody Abs-9 exhibits significant affinity for SpA5.

### Abs-9 provided prophylactic protection against *S. aureus* infection

**Figure 4:**
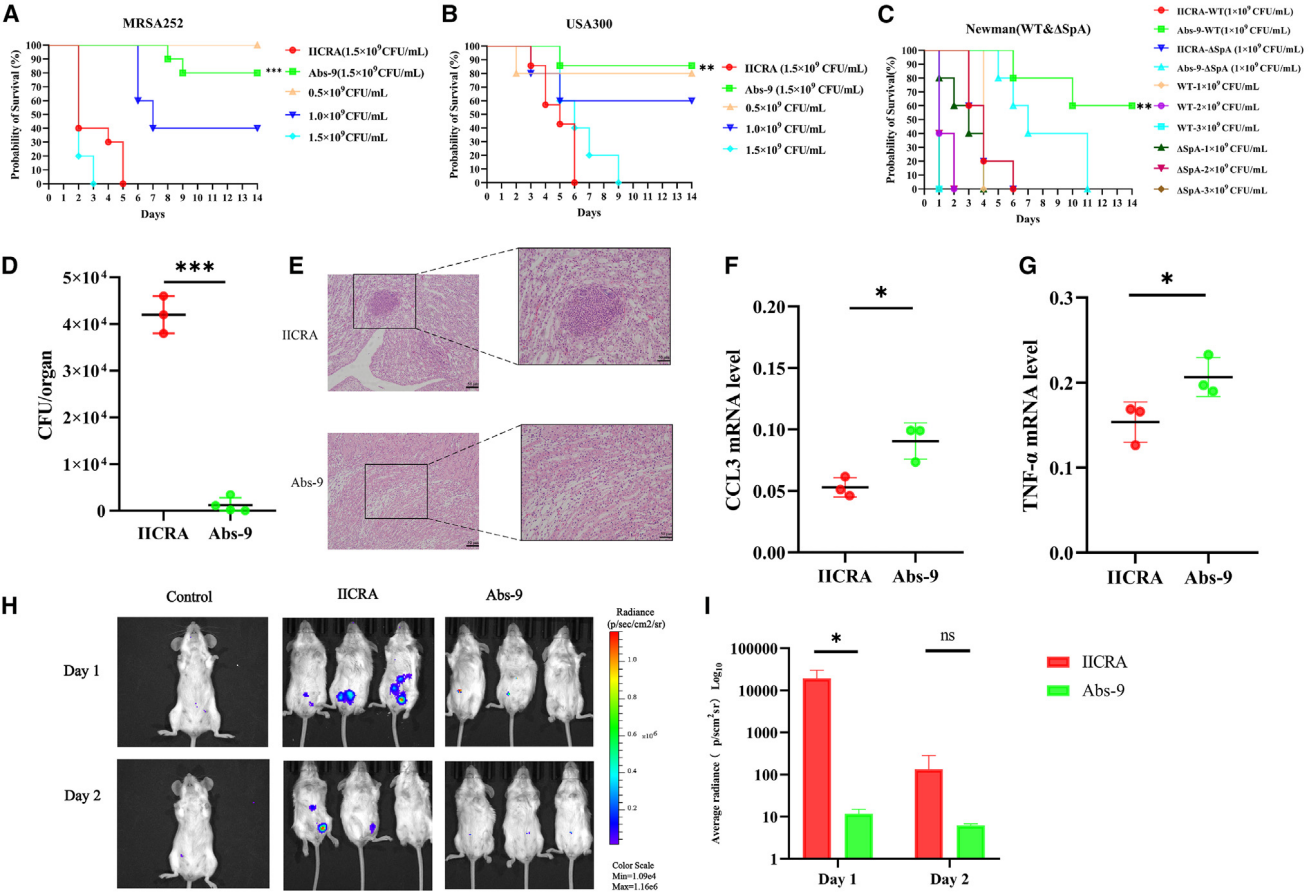
Attenuation of septicemia model in mice through prophylactic administration of antibody Abs-9 (A) Survival rate of mice following MRSA252 strain challenge (*n* = 10). (B) Survival rate of mice following USA300 strain challenge (*n* = 7). (C) Survival rate of following NEWMAN strain challenge (*n* = 5). (D) Calculation of renal bacterial load in mice of the PBS group and Abs-9 group (*n* = 4) three days after infection. Statistical significance was analyzed by Student’s t test. Data are represented as mean ± SEM. (E) Representative histopathological sections of kidney tissues from the PBS group and Abs-9 group (*n* = 5), magnified at 200×, with arrows indicating abscesses in the kidneys. Scale bar, 50 μm. (F) Changes in CCL3 in the kidneys of mice from the PBS group and Abs-9 group (*n* = 3) 3 days after infection. Presented are representative outcomes from one of three independent experiments. Statistical significance was analyzed by Student’s t test. Data are represented as mean ± SEM. (G) Changes in TNF-α in the kidneys of mice from the PBS group and Abs-9 group (*n* = 3) 3 days after infection. Presented are representative outcomes from one of three independent experiments. Statistical significance was analyzed by Student’s t test. Data are represented as mean ± SEM. (H) Bioluminescent image of a representative mouse infected with *S. aureus* Xen29. Images were obtained on the first and second day after infection. Presented are representative outcomes from one of three independent experiments. (I) Quantitative analysis of bioluminescence. (ns, no significance; ∗*p* < 0.05, ∗∗*p* < 0.01, ∗∗∗*p* < 0.001.). Statistical significance was analyzed by Student’s t test. Data are represented as mean ± SEM.

To evaluate the activity of the human antibody Abs-9 *in vivo*, we constructed a mouse sepsis model. We first pre-injected 100 μL (0.8 mg) of the human antibody Abs-9 or isotype control into the tail vein of 6- to 8-week-old BALB/c mice (18–20 g), and at 24 h the mice were injected with different concentrations and different strains of bacteria into the tail vein (MRSA252, USA300, NEWMAN). The results showed that the survival of Abs-9 in the antibody group were 80%, 85.7%, and 60% (Figures 4A–4C), respectively, with statistically significant differences (∗∗∗*p* < 0.001, ∗∗*p* < 0.01) compared with the control group during the 14 consecutive days of observation. This result demonstrated that the human antibody Abs-9 had superior efficacy in the mouse sepsis model, proving its broad-spectrum protective effect against *S. aureus*. Because of the positive effect of this human antibody Abs-9 in preventing *S. aureus* infections, we then injected 0.8 mg of Abs-9 or isotype control antibody into the tail vein of mice infected with a lethal dose of MRSA252 strain 1 h later. However, there was no significant difference in the Abs-9 antibody group, although some mice survived (*p* > 0.05), and thus the results of the experiments were not performed to demonstrate Abs-9 has a significant therapeutic effect against *S. aureus*. In addition, we performed the same survival experiment on the NEWMAN SpA protein knockout strain at the same time as the NEWMAN strain takedown experiment. The results showed a significant protective effect of the antibody group of the wild-type NEWMAN strain compared to the antibody group of the NEWMAN SpA protein knockout over the 14 days of continuous observation. Although, there was a statistically significant difference in the survival time of mice in the NEWMAN SpA knockout antibody group compared to the control group (Figure 4C), and thus the experiments also support the conclusion that the antigen SpA5 is the specific antigen targeted by the antibody Abs-9, as described earlier.

Next, in order to evaluate the prophylactic protection of human antibody Abs-9 against *S. aureus* infection in multiple ways, we used bacterial loading experiments. The MRSA252 takedown dose was adjusted to 0.5 × 10^8^ colony-forming unit (CFU) for tail vein injection, and 72 h later, mouse kidneys were taken and ground to dilute a certain concentration to assess the bacterial load. The results showed that the bacterial load in the kidneys of Abs-9 mice in the antibody group was significantly reduced compared with the control group (*p* < 0.001) (Figure 4D). In addition, histological analysis of H&E staining and tissue sections taken from mouse kidneys after 72 h showed that Abs-9 treatment in the antibody group reduced mouse kidney abscesses compared with the control group (Figure 4E). Both experiments equally demonstrated the significant protective effect of the human antibody Abs-9 against *S. aureus* infection.

In addition, we used fluorescence quantitative PCR experiments to detect the changes of inflammatory factors in the mouse kidney after 72 h. Compared with the control group, the levels of CCL3 and tumor necrosis factor alpha (TNF-α) were significantly higher in the antibody group Abs-9 (∗*p* < 0.05) (Figures 4F and 4G). Finally, we used mice *in vivo* imaging experiment to detect the protective effect of the human antibody Abs-9. The concentration of the fluorescent strain Xen29 was adjusted to 2 × 10^9^ CFU for intraperitoneal injection, and the fluorescence intensity of the mouse peritoneal cavity was observed within two consecutive days. The experimental results showed that compared with the control group, Abs-9 in the antibody group had a significant inhibitory effect on strain Xen29, and the fluorescence values were significantly different within the first day (∗*p* < 0.05) (Figures 4H and 4I). The results of the experiment are similarly indicative of the significant protective effect of the human antibody Abs-9 against *S. aureus* infections. In summary, the human antibody Abs-9 had a significant prophylactic effect against *S. aureus* infection.

### Structural modeling and molecular docking of the human antibody Abs-9 and the antigen SpA5

**Figure 5:**
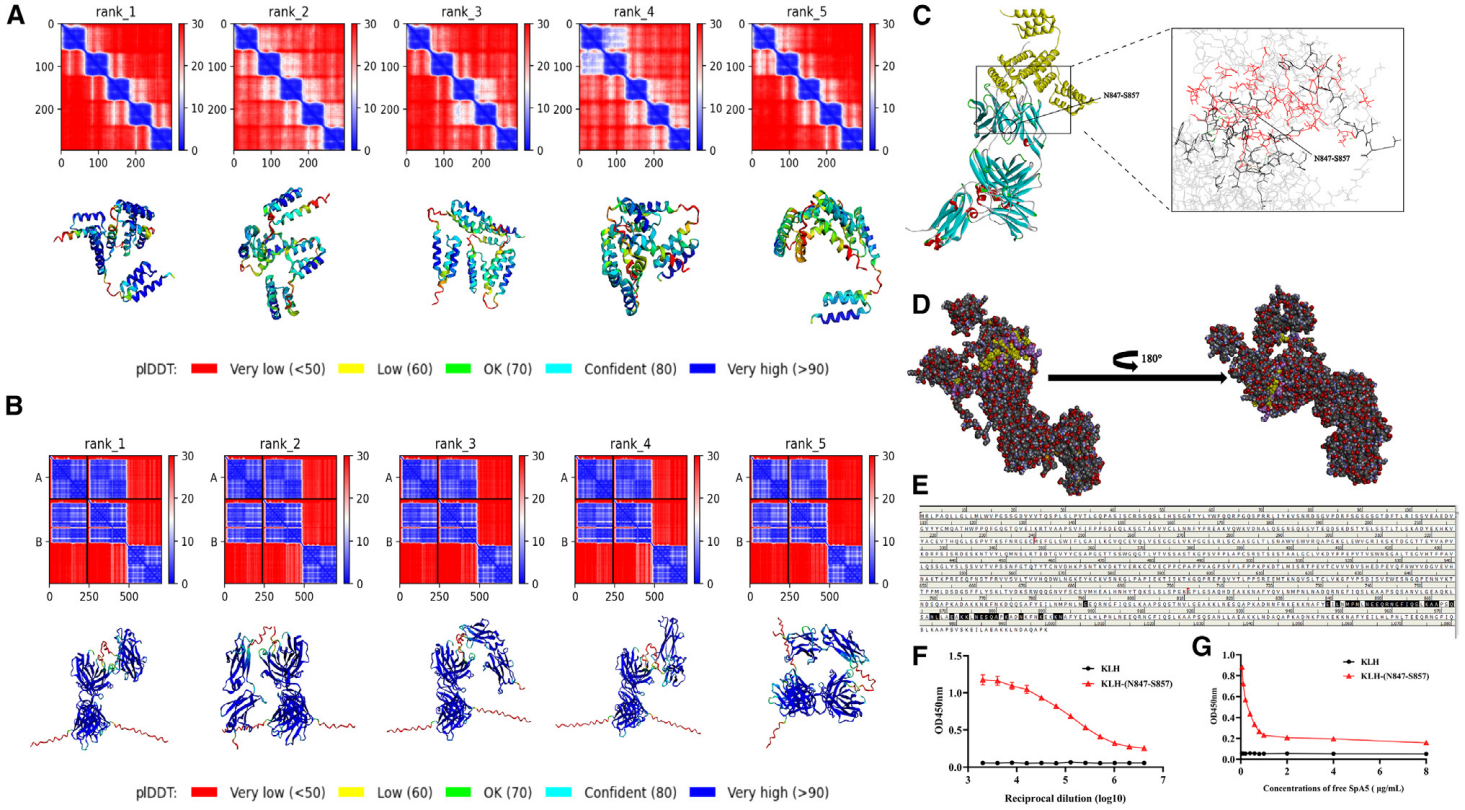
Structure prediction and molecular docking of antigen SpA5 and antibody Abs-9 (A) Structure prediction score plot of antigen SpA5 and its corresponding 3D structure model. (rank1–rank5 are sorted in descending order of PLDD values.). (B) Structure prediction scoring plot for antibody Abs-9 and its corresponding 3D structural model. (rank1–rank5 are sorted in descending order of PLDD values.). (C) 3D structural model of the molecular docking of antigen SpA5 and antibody Abs-9. Abs-9 is shown in blue, red, and green, and SpA5 is shown in yellow. (D) Atomic structure model of the molecular docking of antigen SpA5 and antibody Abs-9. Binding sites on the antigen are shown in yellow and on the antibody in purple. (E) Molecular docking predictions of antigenic epitopes that may bind to antibody Abs-9. (Amino acids marked in black are binding sites). (F) Validation of the affinity of the predicted epitope KLH-(N847-S857) to Abs-9. Data are represented as mean ± SEM. (G) KLH-(N847-S857) and antigen-SpA5-specific competitive inhibition binding antibody Abs-9.

Combining the results of these experiments, we identified the *in vitro* activity of the human antibody Abs-9 as well as its *in vivo* protective effect against a lethal model of *S. aureus* infection, providing a theoretical basis for Abs-9 to become a therapeutic agent for *S. aureus*. However, the mechanism by which Abs-9 exerts its prophylactic-protective effect is unknown. In order to study the binding structure basis between the human antibody Abs-9 and its specific antigen SpA5, first, the 3D theoretical structures of Abs-9 and SpA5 were constructed using the website alphafold2 (Figures 5A and 5B) method. And then, the 3D complex structure of Abs-9 and SpA5 was obtained using molecular docking software deposited in Discovery Studio 2019 program (Figure 5C and 5D). The results showed that the antigenic epitope located on the α-helix structure of SpA5 bound to Abs-9, and the epitopes contained 36 amino acid residues (Figures 5C–5E) (E790, E839, L841, M843, P844, N845, N847, E848, E849, Q850, R851, N852, G853, F854, I855, Q856, S857, K859, A860, A861, Q864, N867, L868, E871, K873, K874, N876, E877, S878, Q879, A880, K882, N885, K889, K892, N893). To validate the binding epitope on SpA5, we coupled keyhole limpet hemocyanin (KLH) to the epitope (N847-S857) and detected good affinity of KLH-(N847-S857) for Abs-9 by ELISA (Figure 5F); furthermore, competitive binding of synthetic peptide N847-S857 and antigen SpA5 to antibody Abs-9 inhibits binding of synthetic peptide to monoclonal antibody (Figure 5G). In conclusion, we modeled the 3D complex structure of the antibody Abs-9 and its specific antigen SpA5 and predicted and validated the epitopes of SpA5. This points out the direction for the subsequent in-depth study of the functional mechanism of antibody Abs-9 and guides the design of *S. aureus* vaccines.

## Discussion

*S. aureus* poses a serious threat to human life due to the problem of drug resistance arising from the misuse of antibiotics.^2^^,^^4^^,^^5^ Therefore, the prevention and treatment of *S. aureus* infections cannot be delayed. Antibodies are effective drugs to neutralize bacterial toxins and can provide timely treatment for infected patients, which is valuable for in-depth research.^9^ In recent years, the antibody drugs Veronate^17^^,^^18^ and tefibazumab^19^^,^^20^^,^^21^^,^^22^^,^^23^ targeting ClfA, pagibaximab^24^^,^^25^^,^^26^ targeting lipophosphatidylcholic acid, and Aurograb^27^^,^^28^ targeting cell membrane proteins were terminated with failure after entering the clinical evaluation. On the other hand, human mAb MEDI4893 and human mAb AR-301, antibody drugs targeting hemolysin and two-component toxins, and Altastaph, an antibody drug targeting podocarp polysaccharides, all showed better results in the clinical evaluations and entered into the next step of clinical evaluation.^29^^,^^30^^,^^31^ Thus, it is necessary for the study of antibodies for the prevention and treatment of *S. aureus* infections for the safety of human life and health.

In light of our findings regarding Abs-9’s efficacy, it is important to discuss the broader context of antibody-mediated immunity against bacterial infections.^32^ *S. aureus* is more common in the skin than in the blood. This suggests that antibody development could be carried out from memory B cells against *S. aureus* virulence factors. Antibody libraries are diverse in their ability to fine-tune antigen specificity through somatic hypermutation, enabling effective responses to invading pathogens. Additionally, a novel protocol for the production of antigen-specific human monoclonal antibodies (hmAbs) developed by Kenneth Smith et al. can rapidly generate numerous antigen-specific hmAbs within a short time.^33^ Based on the aforementioned idea, we used the antibody library constructed by B cell receptor (BCR) sequencing technology for high-throughput sequencing to quickly and effectively screen Abs-9, a highly effective antibody with strong preventive effect. However, apart from clonally enriched B cell clonotypes, the vast majority of sequenced B cells were not utilized in this study and may still be valuable reservoirs for potential hmAb.^14^ Bioinformatics-based selection methods can be used to screen our desired monoclonal antibodies, so we performed structure prediction and molecular docking of the screened human antibody Abs-9 and its specific antigens to find antigenic epitopes that bind to antibody Abs-9. Our study identified Abs-9 as a potent antibody with prophylactic efficacy, targeting specific antigenic epitopes. These findings suggest that using such epitopes, we can screen for additional human antibodies, which may significantly aid in rational vaccine design.

In addition, the recombinant five-component *S. aureus* vaccine (rFSAV), which was developed by the Engineering Center in its previous research on the immune control against *S. aureus*, has shown good results in both phase I and phase II and has now entered phase III clinical trials. In this study, peripheral blood lymphocytes from recombinant *S. aureus* vaccine clinical phase I subjects were used as the main samples to sort out the specific memory B cells binding to five vaccine antigens, and the antibody library constructed by BCR sequencing was used to screen out Abs-9, a human antibody against *S. aureus* with prophylactic efficacy. Abs-9 targeted the pentameric form of *S. aureus* protein A (SpA5) and was found to have good affinity for the antigen SpA5 by ELISA. The antibody Abs-9 was characterized by ELISA, molecular interactions, and mass spectrometry as having good affinity for the antigen SpA5. Evaluation of the mouse sepsis model indicated that Abs-9 had a prophylactic protective effect against *S. aureus* infection and upregulating the levels of CCL3 and TNF-α. Finally, the potential epitopes were identified based on alphafold2 and molecular docking and show the antigenic epitope (N847-S857) that bind to the antibody Abs-9, providing important experimental data to guide the design of vaccines based on antibody structural.

The germline conformation of the antibody-Abs-9-expressing germline VH3-48 gene was screened after immunization with the five-component *S. aureus* vaccine in this study. A previous study by Schneewind et al. used a monoclonal antibody obtained by immunizing mice with nontoxigenic protein A (SpAKKAA) to show binding activity to V_H_3^+^ Fab and inhibits abscess formation.^34^ Our results are a reaffirmation of their research.

In conclusion, the present study successfully screened the human antibody Abs-9 whose antigen is SpA5 for the prevention of *S. aureus* infection and predicted and validated the structure of the binding between the human antibody Abs-9 and its specific antigen SpA5, which provides the possibility of further clinical development and application.

### Limitations of the study

In addition, there are some shortcomings in this study: (1) the inability to accurately separate depleted memory B cells from non-depleted memory B cells in this study could reduce the accuracy of the screening results, (2) the experiment did not systematically analyze antibody-presenting B cells, which may have increased the false-positive rate of non-specific antigen binding during the antigen enrichment step.^8^^,^^14^

## Resource availability

### Lead contact

Further information and requests for resources and reagents should be directed to and will be fulfilled by the lead contact, Bin Wang (wangbin@tmmu.edu.cn).

### Materials availability

All reagents generated in this study are available from the lead contact with a completed materials transfer agreement.

### Data and code availability

• The data supporting the findings of this study are available within the article and its supplemental information. Single-cell sequencing BCR data have been deposited in the NCBI Sequence Read Archive (SRA) and are publicly available as of the date of publication. Accession numbers are listed in the key resources table.
• This paper does not report original code.
• Any additional information required to reanalyze the data reported in this paper is available from the lead contact upon request.

## Acknowledgments

The work was supported by 10.13039/501100001809National Natural Science Foundation of China (31970877, 3220771) and Sichuan Science and Technology Program (2021Y10191).

## Author contributions

The study was conceptualized by Q.Zou. and Q.Zuo. Experiments were conducted and data were analyzed by B.W., X.L., Y.O., J.Z., Y.G., B.L., Y.Z., H.Z., Y.D., Y.W., and R.Z. Project management was handled by W.W., B.W., and X.L. The results were analyzed, and the manuscript was written by W.W., B.W., X.L., and Y.O. All contributing authors have reviewed and endorsed the manuscript.

## Declaration of interests

The authors declare no competing interests.

## STAR★Methods

### Key resources table

**Table:** 

REAGENT or RESOURCE	SOURCE	IDENTIFIER
**Antibodies**		

Goat anti-Human IgG Fc Secondary Antibody, HRP	Thermo Fisher Scientific	Cat#A18817;RRID:AB_2535594
APC/Cy7 anti-human CD56	Biolegend	Cat#318332;RRID:AB_10896424
APC/Cy7 anti-human CD14	Biolegend	Cat#301820;RRID:AB_493695
APC/Cy7 anti-human CD3	Biolegend	Cat#300426;RRID:AB_830755
FITC anti-human CD19	Biolegend	Cat#302206;RRID:AB_314236
PE anti-human CD20	Biolegend	Cat#302306;RRID:AB_314254
APC anti-human IgG Fc	Biolegend	Cat#409306;RRID:AB_11149491

**Bacterial and virus strains**		

Strains: USA300	Third Military Medical University	N/A
Strains: NEWMAN(WT)	Third Military Medical University	N/A
Strains: NEWMAN ΔSpA	Third Military Medical University	N/A
Strains: Xen29	Third Military Medical University	N/A
Strains: MRSA252	Third Military Medical University	N/A

**Chemicals, peptides, and recombinant proteins**		

Polyethylenimine (PEI)	Polysciences Inc.	Cat# 23966-100
EZ-Link Sulfo-NHS-LC-Biotin	Thermo Fisher Scientific	Cat#A39257

**Critical commercial assays**		

PAGE gel rapid preparation kit	Yamei	Cat#PG113
TB Green Premix Ex Taq™ II qPCR	TaKaRa	Cat#CN830A
PrimeScript™ RT Reagent Kit	TaKaRa	Cat# RR037Q

**Deposited data**		

single-cell sequencing of BCR data	This paper	Sequence Read Archive (SRA): PRJNA1187460

**Experimental models: Cell lines**		

Cells: 293F	Third Military Medical University	N/A

**Experimental models: Organisms/strains**		

Mouse: BALB/C	Beijing Vital River Laboratory Animal Technology Co, Ltd	N/A

**Software and algorithms**		

R	https://www.r-project.org	N/A
GraphPad Prism	https://www.graphpad.com/scientificsoftware/prism/	N/A
Discovery Studio 2019	http://www.discoverystudio.net/	N/A
AlphaFold2	https://github.com/deepmind/alphafold	N/A
CellRanger	https://support.10xgenomics.com/single-cell-gene-expression/software/pipelines/latest/installation	N/A
ggplot2	https://cran.r-project.org/	N/A
Customised computer code,see Data S3	This paper	N/A

### Experimental model and study participant details

#### Animals

Female BALB/C mice (6–8 weeks old, 18–20 g) were obtained from Beijing Vital River Laboratory Animal Technology Co, Ltd. (Beijing, People’s Republic of China) and housed under specific pathogen-free (SPF) conditions. The mice were maintained at a constant temperature with an alternating 12 h light/dark cycle. Food and water were available *ad libitum*. The mice were divided into two experimental groups: the isotype control (IICRA) group and the Abs-9 group. All animal experiments were approved by the Animal Ethics Committee of the Army Medical University and were conducted in compliance with all relevant ethical regulations for animal testing and research (AMUWEC2019395).

#### Bacterial strains

Bacterial strains used in this study were incubated overnight on tryptic soy agar (37°C), followed by culture in tryptic soy broth (TSB) for 16 h (37°C, 220 rpm). A 200 μL aliquot of the bacterial solution was re-cultured in 20 mL fresh TSB for 5 h, washed, and appropriately diluted in sterile phosphate-buffered saline (PBS). The optical density at 600 nm (OD_600_) was determined using a spectrophotometer.^35^ The bacterial strains were cultured under these conditions to ensure optimal growth and activity for subsequent *in vivo* infection experiments.

#### Clinical sample collection

This study included 64 healthy volunteers who were vaccinated with recombinant five-component S. aureus vaccine**.** We randomly selected a total of 64 participants from those vaccinated with low-dose (60 μg) and medium-dose (120 μg) vaccines during the phase 1 trial. Among these, samples were chosen from 32 volunteers in each of the low-dose and medium-dose groups. Samples from the high-dose group (240 μg) were utilized in other experiments and thus were not included in this selection. Clinical data for these volunteers are outlined in Table S1. The lymphocytes were isolated from the blood samples of volunteers, followed by BCR sequencing.

#### Additional resources

The study received approval from the Ethics Committee of the Army Medical University, and written informed consent was obtained from the volunteers. The trials are registered with ClinicalTrials.gov, numbers NCT02804711 (phase 1a trial, https://clinicaltrials.gov/search?cond=Staphylococcus%20Aureus&term=NCT02804711) and NCT03966040 (phase 1b trial, https://clinicaltrials.gov/search?cond=Staphylococcus%20Aureus&term=NCT03966040).

#### Cell lines

The 293F cell line was purchased from Chongqing Biaoyue Biotechnology Co., Ltd (Chongqing, China). 293F cells were cultured in OPM-293 CD05 medium (OPM, China) and incubated at 37°C on a constant temperature shaker table in a 5% CO2 incubator. The 293F cell line was stored at −80°C in OPM-293 CD05 medium: DMSO = 9:1 (DMSO, Sigma-Aldrich).

### Method details

#### PBMC isolation

Blood samples from Phase I clinical trial volunteers were diluted with an equal volume of PBS buffer and carefully layered over Ficoll separation solution. After centrifugation at 2000 rpm for 20 min, the white cell layer was collected, washed with PBS, and centrifuged at 1500 rpm for 10 min to remove the supernatant. The cells were resuspended in RPMI medium containing 10% FBS, counted, and cryopreserved. Cell suspensions (1 × 10^7^ cells/mL) were frozen in 1 mL aliquots in cryopreservation tubes, stored at −80°C overnight, and subsequently transferred to liquid nitrogen for long-term storage. In addition, the gene sequences of all five vaccine components are placed in the Data S1.

#### Preparation of biotin-coupled proteins

Sulfo-NHS-LC-Biotin (Thermo Fisher) was mixed with antigen protein solutions and incubated at room temperature for 30 min. The samples were purified using pre-treated Zeba desalting spin columns (Thermo Fisher) according to the manufacturer’s instructions.

#### Flow cytometry sorting

Frozen PBMCs were thawed, incubated for 12 h, and blocked with 5% rat serum. Biotinylated antigenic protein SpA5 was incubated with PBMCs at 4°C for 25 min in the dark, followed by flow cytometric staining. Single antigen-specific memory B lymphocytes were sorted using the gating strategy CD19^+^CD20^+^IgG^+^CD3^−^CD14^−^CD56^−^ (Biolegend). The sorted cells were collected into 20% DMEM medium, kept on ice, and used for downstream experiments within 30 min.

#### BCR sequencing of specific memory B cells

Cell count and viability was estimated using fluorescence Cell Analyzer (Countstar Rigel S2) with AO/PI reagent and then debris and dead cells removal was decided to be performed or not (Miltenyi 130-109-398/130-090-101). Finally fresh cells were washed twice in the RPMI 1640 and then resuspended at 1 × 106 cells per mL in 1×PBS and 0.04% bovine serum albuminat.

Single-cell RNA-Seq libraries were prepared using Chromium Single Cell Human BCR Amplification Kit. Briefly, appropriate number of cells were mixed with reverse transcription reagent and then added to the sample well. Subsequently Barcoded Hydrogel Beads and partitioning oil were dispensed into corresponding wells separately in Chip S3. After emulsion droplet generation reverse transcription were performed at 42°C for 90 min and inactivated at 85°C for 5 min. Next, cDNA was purified from broken droplet and amplified in PCR reaction. The amplified cDNA product was then cleaned, fragmented, end repaired, A-tailed and ligated to sequencing adaptor. Finally the indexed PCR were performed to amplified the DNA representing 3′ poly A part of expressing genes which also contained Cell Barcode and Unique Molecular Index. The indexed sequencing libraries were cleanup with SPRI beads, quantified by quantitative PCR (KAPA Biosystems KK4824) and then PE150 paired-end sequencing was performed using the Illumina platform (10X Genomics).

#### Plasmid expression vector construction

The pcDNA3.1(+) vector (constructed by Genscript Biotech Corporation) was cloned with the BCR sequences obtained from single-cell sequencing at NotI/XbaI restriction sites to create expression vectors for fully human antibodies. The vectors were transformed into TOP10 Escherichia coli strains, which were subsequently cultured in LB medium containing ampicillin for amplification. Plasmids were extracted for further analysis.

#### Expression and purification of human antibody

The concentration of cultured 293F cells was adjusted to the appropriate concentration (10^6^ cells/mL), and the mixture of heavy chain (0.5 μg/mL), light chain (0.67 μg/mL), PEI (2.3 μg/mL) and medium incubated at 37°C for 15 min was added to the 293F cell culture medium, and cultured at 37°C in a constant temperature shaking table of 5% CO_2_ incubator for 5 days. Centrifuge at 3000 g for 30 min at room temperature and collect supernatant. 5 mL IgG Protein A beads (Beyotime) were added into the affinity chromatography column and washed three times with PBS. The transfection supernatant fully combined with Protein A beads packing overnight was added to the chromatographic column for elution. Then add 20 mL of Protein A IgG Binding buffer (Thermo Scientific) eluting beads and repeat. 1 mL 1 M Tris HCI (Solarbio) was added to a 50 mL ultrafiltration tube, and then 10 mL IgG Elution buffer (Thermo Scientific) was added to the chromatographic column. The eluate was collected in an ultrafiltration tube and concentrated to 0.5 mL by centrifugation at 4°C, 3000 rpm. Add 10 mL of PBS and concentrate again. The protein content of ultrafiltration concentrate was detected and stored at −80°C.

#### SDS-PAGE

SDS-PAGE gel was prepared according to the instructions of PAGE gel rapid preparation kit (Yamei, China). The purified antibody was 20 μg adjusted to 24 μL with ultra-pure water, divided into two parts and added with 3μL protein loading buffer (non-reduced) and 3μL protein loading buffer (reduced). The above antibodies were heated in a metal bath at 90°C for 10min. Add 10 μL heated protein into the swim lane, and add 5 μL Marker into the Marker hole. Turn on the electrophoresis apparatus, adjust the voltage to 80 V, electrophoresis until the sample strip is separated, adjust the voltage to 120 V, and turn off the power when it reaches 1 cm away from the bottom of the PAGE rubber. Take out the PAGE glue, put it in the dyeing box, add instantaneous blue dye, and dye it at room temperature for 30 min. Discard the instant blue dye, add grade I water to wash, decolorize at room temperature on the shaking bed, take out the PAGE glue and record the image.

#### ELISA

The antigens HLA, IsdB, mSEB, MntC and SpA5 were diluted into 2 μg/mL with coated solution, 100 μL per well, and added to the ELISA plate at 4°C overnight. Wash the plate once (3 cycles, each cycle 300 μL), add 200 μL sealing liquid to each hole, and seal it at 37°C for 2 h. The purified monoclonal antibodies were diluted to 2 μg/mL with PBST (PBS buffer added with 20% Tween 20). Each positive serum was used as positive control, and PBST was diluted 100 times, and PBST was used as negative control. The diluted monoclonal antibody was added to the first row of the coated ELISA plate at 100 μL/well, then diluted in half to the 12th row successively, and the diluted positive control, negative control and blank control were added to the corresponding well according to the sample addition template, 100 μL/well. Incubation at 37°C for 1 h. The board was washed once, and the Goat Anti-Human IgG Fc (HRP, Abcam) was diluted with PBST at the rate of 1:10 000, 100 μL/well, and incubated at 37°C for 45 min. Wash the board once, add TMB color developing solution 100 μL to each hole, place it at 37°C away from light for 5 min, and add 100 μL termination solution to terminate. The OD_450_ value was detected by a microplate reader.

#### Binding analysis using biolayer interferometry (BLI)

The affinity between antigen and antibody was determined using Octet RED96 system (ForteBio) by Bio-layer Interferometry (BLI). This experiment was performed at room temperature. All protein samples were diluted in PBS containing 0.01% Tween 20. The biosensor was pre-equilibrated in buffer for 600 s. The human antibody at a concentration of 8 μg/mL was loaded on the biosensor for 200 s and flowed with a double dilution of the antigen SpA5 (diluted from 53 μM to 6.63 μM) for 600 s. The probe was then immersed in the buffer for 600 s to measure dissociation. At the end of each reaction, the binding and dissociation states of the antibodies could be easily evaluated and the dissociation equilibrium constants were calculated using Data Analysis 7.0 software.

#### Mass spectrometry

15 mL of MRSA252 was activated according to the above bacterial culture, ultrasonically fragmented and centrifuged, the supernatant was taken and incubated with 200 μL of antibody Abs-9 (8 mg/mL) overnight, and then combined with 1mL of Protein A Beads for 2 h on the next day, then add 1 mL of Protein A IgG Binding buffer (Thermo Scientific) eluting beads and repeat. 0.1 mL 1 M Tris HCI (Solarbio) was added to a 1.5 mL tube, and then 1 mL IgG Elution buffer (Thermo Scientific) was added to the chromatographic column. and the eluent was collected and used for mass spectrometry detection.

#### Establishment of lethal sepsis infection model

Female BALB/c mice were injected into the tail vein with 100 μL of various concentrations of strains. Mouse survival conditions were recorded, and continuous observation was conducted for 14 days.

#### Experiment of survival

Female BALB/c mice were divided into two groups and injected with 100 μL of 0.8 mg/mL Purified Human IgG1 Isotype Control Recombinant Antibody (IICRA) or Abs-9 through the tail vein. After 24 h, each group was injected with 100 μL strains through the tail vein, and the survival of the mice was monitored.

#### Bacterial load of kidney

Female BALB/c mice were divided into two groups and injected with 100 μL of 0.8 mg/mL IICRA or Abs-9 through the tail vein. After 24 h, each group was injected with 100 μL strains through the tail vein. 96 h later, mouse kidneys were collected, homogenized, diluted, and cultured on solid medium for bacterial load assessment, bacterial burden was analyzed using t test.

#### Histological analysis

Mouse kidney tissues were collected, fixed, and processed for histological analysis to observe pathological changes (34, 35).

#### qRT-PCR

RNA reverse transcription and quantitative PCR were performed to analyze gene expression levels in kidney tissues (35) and the results was analyzed using t test.

#### Mice *in vivo* imaging

Female BALB/c mice were divided into two groups and injected with 0.8 mg/mL Purified Human IgG1 Isotype Control Recombinant Antibody or Abs-9 (0.8 mg/mL) through the tail vein. The dose of fluorescent strain Xen29 was adjusted to 2 × 10^10^ CFU/mL for intraperitoneal injection of 100 μL, and the fluorescence intensity in the abdominal cavity of mice was observed continuously for 2 days.

#### Molecular docking

The amino acid sequences of the antibody IgG-9 and the antigen SpA5 were converted into FASTA format according to the format required for 3.7 AlphaFold2. Perform multiple sequence comparisons using tools such as HHblits, Jackhmmer, etc. to provide information on the evolution of the sequences. Download the latest versions of the BFD, Uniref. 90, UniClust30 databases and set up the correct paths for AlphaFold 2 to use to generate multiple sequence alignment (MSA).

Set up the multimer model for structure prediction of multimeric proteins. Set up bfd database path, uniclust30 database path, and pdb70 database path for sequence search, template search, and MSA generation. Use pre-calculated MSAs, skip the MSA generation step and use the provided MSAs directly. input the antibody IgG-9 and SpA5 amino acid sequences and run AlphaFold 2.

Import the PDB files of the predicted IgG-9 antibody and SpA5 antigen in Discovery Studio 2019 software. Optimise the imported structures using Discovery Studio 2019’s cleanup tool. Set the IgG-9 antibody as the receptor, the SpA5 protein as the ligand, and the spatial rotation pairing angle to 15, limit the simulated antigen-antibody binding site to the CDR3 region of the antibody, exclude other negative binding sites, and run the Discovery Studio 2019 Binding Site Qualification Tool program. Import the top 3 scored sites in the CDR3 region of the filtered antibody into the program, and run the Flexible Optimisation Tool program of Discovery Studio 2019. Import the highest scoring binding site after flex optimisation into the program and run the Interaction Analysis Tool program for analysis.

#### Validation of the predicted epitope

Keyhole limpet haemocyanin (KLH) was coupled to the epitope and then diluted to 2 μg/mL with coated solution, 100 μL per well, and added to the ELISA plate at 4°C overnight. Wash the plate once (3 cycles, each cycle 300 μL), add 200 μL sealing liquid to each hole, and seal it at 37°C for 2 h. The purified monoclonal antibody Abs-9 were diluted to 2 μg/mL with PBST. The antigen SpA5 was used as positive control, and PBST was diluted 100 times, and KLH was used as a negative control. The diluted monoclonal antibody was added to the first row of the coated ELISA plate at 100 μL/well, then diluted in half to the 12th row successively (Parallel control wells were set up with 4 rows of KLH-(N847-S857), in two of which the antigen SpA5 was multiply diluted starting at 1:2 for a total of 10 gradients. Antibody Abs-9 was diluted at 1:1000 and mixed with the above diluted antigen in equal volume (50 μL:50 μL) for antigen-antibody specific competitive inhibition reaction. as well as negative control wells), and the diluted positive control, negative control and blank control were added to the corresponding well according to the sample addition template, 100 μL/well. Incubation at 37°C for 1 h. The board was washed once, and the Goat Anti-Human IgG Fc (HRP, Abcam) was diluted with PBST at the rate of 1:10 000, 100 μL/well, and incubated at 37°C for 45 min. Wash the board once, add TMB color developing solution 100 μL to each hole, place it at 37°C away from light for 5 min, and add 100 μL termination solution to terminate. The OD_450_ value was detected by a microplate reader.

### Quantification and statistical analysis

All data were processed and analyzed using Excel and GraphPad prism tailed Student’s t test, one-way-ANOVA and two-way ANOVA. All experiments were repeated at least twice with three replicates involved. Statistical significance is indicated with asterisks as follows: ns, no significance; ∗, *p* < 0.05; ∗∗, *p* < 0.01; ∗∗∗, *p* < 0.001.

## Supplemental information

Document S1. Table S1

Data S1. Nucleotide sequence and amino acid sequence of a five-component *Staphylococcus aureus* vaccine (HI, MntC, SpA5, mSEB). related to Figure 1 and Figure 3 Data S2. Nucleotide sequence and amino acid sequence of antibody Abs-9, related to Figure 3 Data S3. Customized computer code used in the bioinformatics analysis, related to STAR Methods.
