# Supplementary material for: Potent human antibodies against SpA5 identified by high-throughput single-cell sequencing of phase I clinical volunteers’ B cells
Source: iScience. 2024 Dec 18;28(1):111627. doi: 10.1016/j.isci.2024.111627 (PMC11743104; doi:10.1016/j.isci.2024.111627)
Supplement: Document S1. Table S1 [file mmc1.pdf]

iScience, Volume *28*

Supplemental information

Potent human antibodies against SpA5 identified by high-throughput single-cell sequencing of phase I clinical volunteers' B cells

Wenhao Wang, Xin Li, Yangxue Ou, Jinrui Zhou, Yaru Gu, Bixia Liu, Yan Zheng, Ying Wang, Rui Zhang, Quanming Zou, Qianfei Zuo, and Bin Wang

Supplemental table

Table S1. Characteristics of volunteers included in this study.

dose	volunteers number	Age	Sex(F/M)
60 μg	L1	20	F
60 μg	L2	18	F
60 μg	L3	23	M
60 μg	L4	65	F
60 μg	L5	40	F
60 μg	L6	32	F
60 μg	L7	32	F
60 μg	L8	28	F
60 μg	L9	29	F
60 μg	L10	19	M
60 μg	L11	45	M
60 μg	L12	23	F
60 μg	L13	28	F
60 μg	L14	27	F
60 μg	L15	38	M
60 μg	L16	41	F
60 μg	L17	42	M
60 μg	L18	32	M
60 μg	L19	36	M
60 μg	L20	47	M
60 μg	L21	60	M
60 μg	L22	47	M
60 μg	L23	39	M
60 μg	L24	36	M
60 μg	L25	30	F
60 μg	L26	44	F
60 μg	L27	36	M
60 μg	L28	25	M
60 μg	L29	28	M
60 μg	L30	24	F
60 μg	L31	36	M
60 μg	L32	33	F
120 μg	M1	24	M
120 μg	M2	25	M
120 μg	M3	45	M
120 μg	M4	47	F
120 μg	M5	43	M
120 μg	M6	28	M
120 μg	M7	22	M

120 µg	M8	36	M
120 µg	M9	35	F
120 µg	M10	38	F
120 µg	M11	50	M
120 µg	M12	48	F
120 µg	M13	44	F
120 µg	M14	33	F
120 µg	M15	38	F
120 µg	M16	27	F
120 µg	M17	31	M
120 µg	M18	48	F
120 µg	M19	25	F
120 µg	M20	55	M
120 µg	M21	47	F
120 µg	M22	35	M
120 µg	M23	46	F
120 µg	M24	29	F
120 µg	M25	33	F
120 µg	M26	24	F
120 µg	M27	27	F
120 µg	M28	49	F
120 µg	M29	56	F
120 µg	M30	55	M
120 µg	M31	24	F
120 µg	M32	28	M
