# Supplementary material for: Potent human antibodies against SpA5 identified by high-throughput single-cell sequencing of phase I clinical volunteers’ B cells
Source: iScience. 2024 Dec 18;28(1):111627. doi: 10.1016/j.isci.2024.111627 (PMC11743104; doi:10.1016/j.isci.2024.111627)
Supplement: Data S1. Nucleotide sequence and amino acid sequence of a five-component Staphylococcus aureus vaccine (HI, MntC, SpA5, mSEB). related to Figure 1 and Figure 3 — Data S2. Nucleotide sequence and amino acid sequence of antibody Abs-9, related to Figure 3 Data S3. Customized computer code used in the bioinformatics analysis, related to STAR Methods. [file mmc2.zip › supplemental data/Data S1.docx]

**Data S1.**

HI (Hla-IsdB): nucleotide sequence:

GGATCCATGGCAGATTCTGATATTAATATTAAAACCGGTACTACAGATATAGGAAGCAATACTACAGTAAAAACAGGTGATTTAGTCACTTATGATAAAGAAAATGGCATGCTGAAAAAAGTATTTTATAGTTTTATCGATGATAAAAATCACAATAAAAAAATATTAGTCATCAGAACGAAAGGTACCATTGCTGGTCAGTATAGAGTTTATAGCGAAGAAGGCGCTAATAAAAGCGGTTTAGCCTGGCCTTCAGCATTTAAGGTACAGTTGCAACTACCTGATAATGAAGTAGCACAAATTTCTGATTACTACCCTAGAAATTCAATTGATACTAAAGAATATATGAGTACATTAACTTATGGATTCAACGGTAACGTGACTGGTGATGATTCTGGAAAAATTGGCGGTTTGATTGGTGCAAATGTTTCAATTGGTCATACACTCAAATATGTTCAACCAGATTTCAAAACAATTTTAGAAAGTCCAACTGATAAAAAAGTAGGTTGGAAAGTTATATTTAACAATATGGTGAATCAAAATTGGGGACCATATGACAGAGATTCTTGGAACCCAGTATATGGCAATCAACTTTTCATGAAAACAAGAAACGGTTCAATGAAAGCAGCAGAGAACTTCCTTGACCCTAACAAAGCAAGTTCTTTATTATCTTCAGGATTCTCACCAGACTTTGCTACAGTTATTACTATGGATAGGAAAGCAACCAAACAACAAACAAATATAGATGTAATATACGAACGAGTTCGTGATGACTATCAACTACATTGGACTTCAACAAATTGGAAAGGTACCAATACTAAAGATAAATGGACAGATCGTTCTTCAGAAAGATATAAAATCGATTGGGAAAAAGAAGAAATGACAAATGGTGGCGGTGGCAGCAAAATGACCGATTTACAAGATACAAAATATGTTGTTTATGAAAGTGTTGAGAATAACGAATCTATGATGGATGCTTTTGTTAAACACCCTATTAAAACAGGTATGCTTAATGGCAAAAAATATATGGTAATGGAAACTACTAATGACGATTACTGGAAAGATTTCATGGTTGAAGGTCAACGTGTTAGAACAATTAGCAAAGATGCTAAAAATAACACTAGAACGATTATCTTCCCATATGTTGAAGGTAAAACTCTATATGATGCTATCGTTAAAGTTCACGTAAAAACGATTGATTATGATGGACAATACCATGTCAGAATCGTTGATAAAGAAGCATTTACTAAAGCCAATCTCGAGTGATAAGCGGCCGC

HI：(Hla-IsdB): amino acid sequence:

GSMADSDINIKTGTTDIGSNTTVKTGDLVTYDKENGMLKKVFYSFIDDKNHNKKILVIRT
KGTIAGQYRVYSEEGANKSGLAWPSAFKVQLQLPDNEVAQISDYYPRNSIDTKEYMSTLT
YGFNGNVTGDDSGKIGGLIGANVSIGHTLKYVQPDFKTILESPTDKKVGWKVIFNNMVNQNWGPYDRDSWNPVYGNQLFMKTRNGSMKAAENFLDPNKASSLLSSGFSPDFATVITMDRKATKQQTNIDVIYERVRDDYQLHWTSTNWKGTNTKDKWTDRSSERYKIDWEKEEMTNGGGGSKMTDLQDTKYVVYESVENNESMMDAFVKHPIKTGMLNGKKYMVMETTNDDYWKDFMVEGQRVRTISKDAKNNTRTIIFPYVEGKTLYDAIVKVHVKTIDYDGQYHVRIVDKEAFTKANLE

MntC:nucleotide sequence:

GGATCCAGCAGTGATAAGTCAAATGGCAAACTAAAAGTAGTAACGACGAATTCAATTTTATATGATATGGCTAAAAATGTTGGTGGAGACAACGTCGATATTCATAGTATTGTACCTGTTGGTCAAGATCCTCATGAATATGAAGTTAAACCTAAAGATATTAAAAAGTTAACTGACGCTGACGTTATTTTATACAACGGATTAAATTTAGAGACTGGTAACGGTTGGTTTGAAAAAGCCTTAGAACAGGCTGGTAAATCATTAAAAGATAAAAAAGTTATCGCAGTATCAAAAGATGTTAAACCTATCTATTTAAACGGTGAAGAAGGCAACAAAGATAAACAAGATCCACACGCATGGTTAAGTTTAGATAACGGTATTAAATACGTAAAAACAATTCAACAAACATTTATCGATAACGACAAAAAACATAAAGCAGATTATGAAAAGCAAGGTAACAAATACATTGCTCAATTGGAAAAATTAAATAACGACAGTAAAGACAAATTTAATGACATTCCAAAAGAACAACGTGCCATGATTACAAGTGAAGGTGCCTTCAAGTACTTCTCAAAACAATACGGTATTACACCAGGTTATATTTGGGAAATTAACACTGAAAAACAAGGTACACCAGAACAAATGAGACAAGCTATTGAGTTTGTTAAAAAGCACAAATTAAAACACTTATTAGTAGAAACAAGTGTTGATAAGAAAGCAATGGAAAGTTTATCTGAAGAAACGAAGAAAGATATCTTTGGTGAAGTGTACACAGATTCAATCGGTAAAGAAGGCACTAAAGGTGACTCTTACTACAAAATGATGAAATCAAATATTGAAACTGTACACGGAAGCATGAAATAATAAGCGGCCGC

MntC: amino acid sequence:

GSSSDKSNGKLKVVTTNSILYDMAKNVGGDNVDIHSIVPVGQDPHEYEVKPKDIKKLTDA
DVILYNGLNLETGNGWFEKALEQAGKSLKDKKVIAVSKDVKPIYLNGEEGNKDKQDPHAWLSLDNGIKYVKTIQQTFIDNDKKHKADYEKQGNKYIAQLEKLNNDSKDKFNDIPKEQRAMITSEGAFKYFSKQYGITPGYIWEINTEKQGTPEQMRQAIEFVKKHKLKHLLVETSVDKKAMESLSEETKKDIFGEVYTDSIGKEGTKGDSYYKMMKSNIETVHGSMK

SpA5: nucleotide sequence:

GGATCCGCGCAACACGATGAAGCTAAAAAAAATGCTTTTTATCAAGTGTTAAATATGCCTAACTTAAACGCTGATCAACGTAATGGTTTTATCCAAAGCCTTAAAGCAGCACCAAGCCAAAGTGCTAACGTTTTAGGTGAAGCTCAAAAACTTAATGACTCTCAAGCTCCAAAAGCTGATGCGAAAAAAAATAAGTTCAACAAAGATCAACAAAGCGCCTTCTATGAAATCTTGAACATGCCTAACTTAAACGAAGAGCAACGCAATGGTTTCATTCAAAGTCTTAAAGCAGCACCAAGCCAAAGCACTAACGTTTTAGGTGAAGCTAAAAAATTAAACGAATCTCAAGCACCGAAAGCTGACAACAATTTCAACAAAGAAAAAAAAAATGCTTTCTATGAAATCTTGAACATGCCTAACTTGAACGAAGAACAACGCAATGGTTTCATCCAAAGCTTAAAAGCAGCACCAAGTCAAAGTGCTAACCTTTTAGCAGAAGCTAAAAAGTTAAATGAATCTCAAGCACCGAAAGCTGATAACAAATTCAACAAAGAAAAAAAAAATGCTTTCTATGAAATCTTACATTTACCTAACTTAAATGAAGAACAACGCAATGGTTTCATCCAAAGCTTAAAAGCAGCACCAAGCCAAAGCGCTAACCTTTTAGCAGAAGCTAAAAAGCTAAATGATGCACAAGCACCAAAAGCTGACAACAAATTCAACAAAGAAAAAAAAAATGCTTTCTATGAAATTTTACATTTACCTAACTTAACTGAAGAACAACGTAACGGCTTCATCCAAAGCCTTAAAGCAGCACCTTCAGTGAGCAAAGAAATTTTAGCAGAAGCTAAAAAGCTAAACGATGCTCAAGCACCAAAATAATGAGCGGCCGC

SpA5: amino acid sequence:

GPLGSAQHDEAKKNAFYQVLNMPNLNADQRNGFIQSLKAAPSQSANVLGEAQKLNDSQAPKADAKKNKFNKDQQSAFYEILNMPNLNEEQRNGFIQSLKAAPSQSTNVLGEAKKLNESQAPKADNNFNKEKKNAFYEILNMPNLNEEQRNGFIQSLKAAPSQSANLLAEAKKLNESQAPKADNKFNKEKKNAFYEILHLPNLNEEQRNGFIQSLKAAPSQSANLLAEAKKLNDAQAPKADNKFNKEKKNAFYEILHLPNLTEEQRNGFIQSLKAAPSVSKEILAEAKKLNDAQAPK

mSEB: nucleotide sequence:

CCATGGAGAGTCAACCAGATCCTAAACCAGATGAGTTGCACAAATCGAGTAAATTCACTGGTTTGATGGAAAATATGAAAGTTTTGTATGATGATAATCATGTATCAGCAATAAACGTTAAATCTATAGATCAATTTCGCTACTTTGACTTAATATATTCTATTAAGGACACTAAGTTAGGGAATTATGATAATGTTCGAGTCGAATTTAAAAACAAAGATTTAGCTGATAAATACAAAGATAAATACGTAGATGTGTTTGGAGCTAATGCGTATTATCAATGTGCGTTTTCTAAAAAAACGAATGATATTAATTCGCATCAAACTGACAAACGAAAAACTTGTATGTATGGTGGTGTAACTGAGCATAATGGAAACCAATTAGATAAATATAGAAGTATTACTGTTCGGGTATTTGAAGATGGTAAAAATTTATTATCTTTTGACGTACAAACTAATAAGAAAAAGGTGACTGCTCAAGAATTAGATTACCTAACTCGTCACTATTTGGTGAAAAATAAAAAACTCTATGAATTTAACAACTCGCCTTATGAAACGGGATATATTAAATTTATAGAAAATGAGAATAGCTTTTGGTATGACATGATGCCTGCACCAGGAGATAAATTTGACCAATCTAAATATTTAATGATGTACAATGACAATAAAATGGTTGATTCTAAAGATGTGAAGATTGAAGTTTATCTTACGACAAAGAAAAAGTAATGAGGATCC

mSEB: amino acid sequence:

PWRVNQILNQMSCTNRVNSLVWKIKFCMMIIMYQQTLNLINFATLTYILLRTLSGIMIMFESNLKTKILINTKINTMCLELMRIINVRFLKKRMILIRIKLTNEKLVCMVVLSIMETNINIEVLLFGYLKMVKIYYLLTYKLIRKRLLKNITLVTIWKIKNSMNLTTRLMKRDILNLKMRIAFGMTCLHQEINLTNLNICTMTIKWLILKMRLKFILRQRKSNED
