# Supplementary material for: Potent human antibodies against SpA5 identified by high-throughput single-cell sequencing of phase I clinical volunteers’ B cells
Source: iScience. 2024 Dec 18;28(1):111627. doi: 10.1016/j.isci.2024.111627 (PMC11743104; doi:10.1016/j.isci.2024.111627)
Supplement: Data S1. Nucleotide sequence and amino acid sequence of a five-component Staphylococcus aureus vaccine (HI, MntC, SpA5, mSEB). related to Figure 1 and Figure 3 — Data S2. Nucleotide sequence and amino acid sequence of antibody Abs-9, related to Figure 3 Data S3. Customized computer code used in the bioinformatics analysis, related to STAR Methods. [file mmc2.zip › supplemental data/Data S2.docx]

**Data S2.**

Heavy chain**:** nucleotide sequence:

GCCACCATGGAATGGTCATGGGTCTTCTTATTTTTCTTAAGTGTTACTACCGGTGTCCACTCCATGGACCTCCTGCACAAGAACATGAAACACCTGTGGTTCTTCCTCCTCCTGGTGGCAGCTCCCAGATGGGTCCTGTCCCAGGTGCAGCTACAGCAGTGGGGCGCAGGACTGTTGAAGCCTTCGGAGACCCTGTCCCTCACCTGCGCTGTCTATGGTGGGTCCTTCAGTGGTTACTACTGGAGCTGGATCCGCCAGCCCCCAGGGAAGGGGCTGGAGTGGATTGGGGAAATCAATCATAGTGGAAGCACCAACTACAACCCGTCCCTCAAGAGTCGAGTCACCATATCGGTAGACACGTCCAAGAACCAGTTCTCCCTGAAGCTGAGCTCTGTGACCGCCGCGGACACGGCTGTGTATTACTGTGCGAGGCGTTCCTACGGTGTCAAACCCTTTGACTACTGGGGCCAGGGAACCCTGGTCACCGTCTCCTCAGCCTCCACCAAGGGCCCATCGGTCTTCCCCCTGGCACCCTCCTCCAAGAGCACCTCTGGGGGCACAGCAGCCCTGGGCTGCCTGGTCAAGGACTACTTCCCCGAACCGGTGACGGTGTCGTGGAACTCAGGCGCCCTGACCAGCGGCGTGCACACCTTCCCGGCTGTCCTACAGTCCTCAGGACTCTACTCCCTCAGCAGCGTGGTGACCGTGCCCTCCAGCAGCTTGGGCACCCAGACCTACATCTGCAACGTGAATCACAAGCCCAGCAACACCAAGGTGGACAAGAAAGTTGAGCCCAAATCTTGTGACAAAACTCACACATGCCCACCGTGCCCAGCACCTGAACTCCTGGGGGGACCGTCAGTCTTCCTCTTCCCCCCAAAACCCAAGGACACCCTCATGATCTCCCGGACCCCTGAGGTCACATGCGTGGTGGTGGACGTGAGCCACGAAGACCCTGAGGTCAAGTTCAACTGGTACGTGGACGGCGTGGAGGTGCATAATGCCAAGACAAAGCCGCGGGAGGAGCAGTACAACAGCACGTACCGTGTGGTCAGCGTCCTCACCGTCCTGCACCAGGACTGGCTGAATGGCAAGGAGTACAAGTGCAAGGTCTCCAACAAAGCCCTCCCAGCCCCCATCGAGAAAACCATCTCCAAAGCCAAAGGGCAGCCCCGAGAACCACAGGTGTACACCCTGCCCCCATCCCGGGATGAGCTGACCAAGAACCAGGTCAGCCTGACCTGCCTGGTCAAAGGCTTCTATCCCAGCGACATCGCCGTGGAGTGGGAGAGCAATGGGCAGCCGGAGAACAACTACAAGACCACGCCTCCCGTGCTGGACTCCGACGGCTCCTTCTTCCTCTACAGCAAGCTCACCGTGGACAAGAGCAGGTGGCAGCAGGGGAACGTCTTCTCATGCTCCGTGATGCATGAGGCTCTGCACAACCACTACACACAGAAGAGCCTCTCCCTGTCTCCGGGTAAA

Heavy chain**:** amino acid sequence:

ATMEWSWVFLFFLSVTTGVHSMDLLHKNMKHLWFFLLLVAAPRWVLSQVQLQQWGAGLLKPSETLSLTCAVYGGSFSGYYWSWIRQPPGKGLEWIGEINHSGSTNYNPSLKSRVTISVDTSKNQFSLKLSSVTAADTAVYYCARRSYGVKPFDYWGQGTLVTVSSASTKGPSVFPLAPSSKSTSGGTAALGCLVKDYFPEPVTVSWNSGALTSGVHTFPAVLQSSGLYSLSSVVTVPSSSLGTQTYICNVNHKPSNTKVDKKVEPKSCDKTHTCPPCPAPELLGGPSVFLFPPKPKDTLMISRTPEVTCVVVDVSHEDPEVKFNWYVDGVEVHNAKTKPREEQYNSTYRVVSVLTVLHQDWLNGKEYKCKVSNKALPAPIEKTISKAKGQPREPQVYTLPPSRDELTKNQVSLTCLVKGFYPSDIAVEWESNGQPENNYKTTPPVLDSDGSFFLYSKLTVDKSRWQQGNVFSCSVMHEALHNHYTQKSLSLSPGK

Light chain: nucleotide sequence:

GCCACCATGGAATGGTCATGGGTCTTCTTATTTTTCTTAAGTGTTACTACCGGTGTCCACTCCATGGCCTGGACCCCTCTCTGGCTCACTCTCTTCACTCTTTGCATAGGTTCTGTGGTTTCTTCTGAGCTGACTCAGGACCCTGCTGTGTCTGTGGCCTTGGGACAGACAGTCAGGATCACATGCCAAGGAGACAGCCTCAGAACCTATTATGCAAGCTGGTACCAGCAGAAGCCAGGACAGGCCCCTGCACTTGTCATCTATGGTAAAAACAACCGGCCCTCAGGGATCCCAGACCGATTCTTTGGCTCCAGCTCAGGAAACACAGCTTCCTTGACCATCACTGGGGCTCAGGCGGAAGATGAGGCTGACTATTACTGTAACTCCCGGGACAGCAGTGGTAACCATCACCCGGTATTCGGCGGAGGGACGAAACTGACCGTCCTAAGTCAGCCCAAGGCTGCCCCCTCGGTCACTCTGTTCCCGCCCTCCTCTGAGGAGCTTCAAGCCAACAAGGCCACACTGGTGTGTCTCATAAGTGACTTCTACCCGGGAGCCGTGACAGTGGCCTGGAAGGCAGATAGCAGCCCCGTCAAGGCGGGAGTGGAGACCACCACACCCTCCAAACAAAGCAACAACAAGTACGCGGCCAGCAGCTATCTGAGCCTGACGCCTGAGCAGTGGAAGTCCCACAGAAGCTACAGCTGCCAGGTCACGCATGAAGGGAGCACCGTGGAGAAGACAGTGGCCCCTACAGAATGTTCA

Light chain: amino acid sequence:

ATMEWSWVFLFFLSVTTGVHSMAWTPLWLTLFTLCIGSVVSSELTQDPAVSVALGQTVRITCQGDSLRTYYASWYQQKPGQAPALVIYGKNNRPSGIPDRFFGSSSGNTASLTITGAQAEDEADYYCNSRDSSGNHHPVFGGGTKLTVLSQPKAAPSVTLFPPSSEELQANKATLVCLISDFYPGAVTVAWKADSSPVKAGVETTTPSKQSNNKYAASSYLSLTPEQWKSHRSYSCQVTHEGSTVEKTVAPTECS
