# Supplementary material for: Potent human antibodies against SpA5 identified by high-throughput single-cell sequencing of phase I clinical volunteers’ B cells
Source: iScience. 2024 Dec 18;28(1):111627. doi: 10.1016/j.isci.2024.111627 (PMC11743104; doi:10.1016/j.isci.2024.111627)
Supplement: Data S1. Nucleotide sequence and amino acid sequence of a five-component Staphylococcus aureus vaccine (HI, MntC, SpA5, mSEB). related to Figure 1 and Figure 3 — Data S2. Nucleotide sequence and amino acid sequence of antibody Abs-9, related to Figure 3 Data S3. Customized computer code used in the bioinformatics analysis, related to STAR Methods. [file mmc2.zip › supplemental data/Data S3.docx]

**Data S3.**

options(stringsAsFactors=F)

library(vegan)

library(ggplot2)

library(RColorBrewer)

library(igraph)

library(dplyr)

library(tidyr)

library(circlize)

library(ggalluvial)

library(ggfittext)

library(cowplot)

##file clones.perCell.txt get from cellranger vdj

result <- read.table("clones.perCell.txt",header=T,sep="\t")

clonotype_result <- unique(result$clonotype) %>% as.data.frame()

colnames(clonotype_result) <- "clonotypes"

ncells <- nrow(result)

clonotype_freq <- function(bcr_clonotype) {

num_cells <- result[result$clonotype == bcr_clonotype,] %>% nrow()

ratio_cells <- num_cells/ncells

return(c(num_cells,ratio_cells))

}

bcr_clonotype_info <- as.data.frame(t(as.data.frame(apply(clonotype_result,1,clonotype_freq))))

colnames(bcr_clonotype_info) <- c("cell number","cell ratio")

bcr_clonotype_info$clonotype <- clonotype_result$clonotypes

bcr_clonotype_info <- subset(bcr_clonotype_info,select=c("clonotype","cell number","cell ratio"))

bcr_clonotype_info <- bcr_clonotype_info[order(bcr_clonotype_info$`cell number`,decreasing=T),]

write.table(bcr_clonotype_info,file="perClonotype.txt",quote=F,col.names=T,row.names=F,sep="\t")

##Fig2A plot the clonotype network

bcr_temp <- bcr_clonotype_info

bcr_temp$from <- bcr_temp$clonotype

bcr_temp$to <- bcr_temp$clonotype

head(bcr_temp)

edges <- subset(bcr_temp,select=c("from","to"))

edges <- edges[which(edges$from %in% bcr_temp$clonotype) ,]

edges <- edges[which(edges$to %in% bcr_temp$clonotype) ,]

a <- 1

b <- bcr_temp[1,2]

if(b == 1) {b <- b + 0.00001}

c <- 1

d <- 5

bcr_temp$size <- c+ (bcr_temp$`cell number`-a)/(b-a)*(d-c)

graph_sample <- graph_from_data_frame(edges,directed=FALSE,vertices=bcr_temp)

graph_sample

pdf("clonotype.network.pdf")

par(mar=c(0,0,0,0)+.1)

plot(graph_sample,vertex.size=bcr_temp$size,edge.color="white",vertex.color="cyan3",vertex.label=NA)

dev.off()

##Fig2B. plot the clonotype Donutchar plot

data <- data.frame()

## Because the input clonotype have been sort based on Frequency

for(i in 1:10) {

index <- 5*i

from <- index-4

count <- sum(bcr_clonotype_info[from:index,]$`cell number`)

Fraction <- sum(bcr_clonotype_info[from:index,]$`cell ratio`)

data_temp <- data.frame(category=paste("Top",paste(from,index,sep="-"),sep=" "),count=count,fraction=Fraction)

data <- rbind(data,data_temp)

}

clonotype_other <- bcr_clonotype_info[51:nrow(bcr_clonotype_info),]

count <- sum(clonotype_other$`cell number`)

Fraction <- sum(clonotype_other$`cell ratio`)

data_temp <- data.frame(category="Others",count=count,fraction=Fraction)

data <- rbind(data,data_temp)

# Compute the cumulative percentages (top of each rectangle)

data$ymax <- cumsum(data$fraction)

# Compute the bottom of each rectangle

data$ymin <- c(0, head(data$ymax, n=-1))

data$category <- factor(data$category,levels=data$category)

# Make the plot

colourCount <- length(unique(data$category))

getPalette <- colorRampPalette(brewer.pal(9, "Set1"))

p <- ggplot(data, aes(ymax=ymax, ymin=ymin, xmin=3,xmax=4,fill=category)) +

scale_fill_manual(values = getPalette(colourCount)) +

theme_bw() +

geom_rect() +

theme_void() +

xlim(c(2, 4)) +

theme(plot.margin=unit(c(0.5,0.5,0.5,1),'lines')) +

theme(legend.title=element_blank()) +

coord_polar(theta="y") # Try to remove that to understand how the chart is built initially

pdf("clones.Donutchar.pdf",width=12,height=8)

p

dev.off()

clonotype <- bcr_clonotype_info %>% tidyr::separate(clonotype,c("IGHV","IGHJ","IGH-CDR3","IGLKV","IGLKJ","IGLK-CDR3"),"[|]")

IGH_Vgene <- unique(clonotype$IGHV)

IGH_Jgene <- unique(clonotype$IGHJ)

clonotype_IGL <- clonotype[grep("IGL",clonotype$IGLKV),]

colnames(clonotype_IGL) <- c("IGHV","IGHJ","IGH-CDR3","IGLV","IGLJ","IGL-CDR3","cell number","cell ratio")

IGL_Vgene <- unique(clonotype_IGL$IGLV)

IGL_Jgene <- unique(clonotype_IGL$IGLJ)

clonotype_IGK <- clonotype[grep("IGK",clonotype$IGLKV),]

colnames(clonotype_IGK) <- c("IGHV","IGHJ","IGH-CDR3","IGKV","IGKJ","IGK-CDR3","cell number","cell ratio")

IGK_Vgene <- unique(clonotype_IGK$IGKV)

IGK_Jgene <- unique(clonotype_IGK$IGKJ)

## function V_J_gene_freq used to compute Vgene or J gene usage frequency

V_J_gene_freq <- function(genelist,type,chain) {

if(chain == "IGL") {

clonotype_temp <- clonotype_IGL

}else if(chain == "IGK") {

clonotype_temp <- clonotype_IGK

}else {

clonotype_temp <- clonotype

}

frequency <- data.frame()

for (i in 1:length(genelist)) {

ratio <- sum(clonotype_temp[clonotype_temp[[type]]==genelist[i],]$`cell ratio`)

frequency[i,1] <- genelist[i]

frequency[i,2] <- ratio

}

colnames(frequency) <- c(type,"Frequency")

frequency <- frequency[order(frequency$Frequency,decreasing=T),]

frequency[[type]] <- factor(frequency[[type]],levels=frequency[[type]])

return(frequency)

}

## V-J pairs of each chain seperately

clonotype$IGH_VJgene <- paste(clonotype$IGHV,clonotype$IGHJ,sep="|")

clonotype_IGL$IGH_VJgene <- paste(clonotype_IGL$IGHV,clonotype_IGL$IGHJ,sep="|")

clonotype_IGL$IGL_VJgene <- paste(clonotype_IGL$IGLV,clonotype_IGL$IGLJ,sep="|")

clonotype_IGK$IGH_VJgene <- paste(clonotype_IGK$IGHV,clonotype_IGK$IGHJ,sep="|")

clonotype_IGK$IGK_VJgene <- paste(clonotype_IGK$IGKV,clonotype_IGK$IGKJ,sep="|")

IGH_VJgene <- unique(clonotype$IGH_VJgene)

IGL_VJgene <- unique(clonotype_IGL$IGL_VJgene)

IGK_VJgene <- unique(clonotype_IGK$IGK_VJgene)

VJ_IGH <- V_J_gene_freq(IGH_VJgene,"IGH_VJgene","IGH")

VJ_IGL <- V_J_gene_freq(IGL_VJgene,"IGL_VJgene","IGL")

VJ_IGK <- V_J_gene_freq(IGK_VJgene,"IGK_VJgene","IGK")

write.table(VJ_IGH,file="IGH.VJ.txt",sep="\t",quote=F,col.names=T,row.names=F)

write.table(VJ_IGL,file="IGL.VJ.txt",sep="\t",quote=F,col.names=T,row.names=F)

write.table(VJ_IGK,file="IGK.VJ.txt",sep="\t",quote=F,col.names=T,row.names=F)

# circos plot

freq2color <- function(value) {

if(value >0.05) {

color <- "red"

}else if(value <=0.05 & value >=0.01) {

color <- "green"

}else if(value <0.01) {

color <- "blue"

}

}

## subfunction for compute V/J gene usage

gene_freq <- function(genelist,type) {

##type should be V or J

result_temp <- data.frame()

for (gene in genelist) {

data_temp <- data[data[[type]]==gene,]

freq_temp <- sum(data_temp$Frequency)

result1 <- data.frame(gene=gene,frequency=freq_temp)

result_temp <- rbind(result_temp,result1)

}

#result_temp <- result_temp[order(result_temp$Frequency,decreasing=T),]

return(result_temp)

}

## circos for IGH, IGK and IGL

circos_plot_input <- function(chain) {

if(chain == "IGH") {

VJ_select <- VJ_IGH

}else if(chain == "IGK") {

VJ_select <- VJ_IGK

}else if(chain == "IGL") {

VJ_select <- VJ_IGL

}

colnames(VJ_select) <- c("VJgene","Frequency")

VJ_select <- VJ_select %>% separate(VJgene,c("V","J"),"[|]")

VJ_select$Color <- apply(as.data.frame(VJ_select$Frequency),1,freq2color)

print(head(VJ_select))

Vgene <- unique(VJ_select$V)

Jgene <- unique(VJ_select$J)

V_freq <- gene_freq(Vgene,"V")

J_freq <- gene_freq(Jgene,"J")

order <- c(V_freq$V,J_freq$J)

colnames(VJ_select) <- c("from","to","value","Color")

VJ_select$from <- factor(VJ_select$from)

VJ_select$to <- factor(VJ_select$to)

#colors <- as.vector(VJ_select$Color)

#VJ_select <- subset(VJ_select,select=c("from","to","value"))

return(VJ_select)

}

for (chain in c("IGH","IGK","IGL")) {

input <- circos_plot_input(chain)

colors <- as.vector(input$Color)

print(colors)

input <- subset(input,select=c("from","to","value"))

pdf(paste(chain,"VJ.circlize.pdf",sep="."),width=20,height=20)

chordDiagramFromDataFrame(input,annotationTrack = "grid",grid.col ="grey",annotationTrackHeight=0.03, col=colors, transparency=0.4)

circos.track(track.index = 1, panel.fun = function(x, y) {

circos.text(CELL_META$xcenter, CELL_META$ylim[1], CELL_META$sector.index,

facing = "clockwise", niceFacing = TRUE, adj = c(-0.5, 0.0),cex=1.5)

}, bg.border = NA)

circos.clear()

dev.off()

}

##for V-J pairs for two chains together

#IGH-V-IGH-J-IGL-V-IGL-J

clonotype_IGL$HLVJ <- paste(clonotype_IGL$IGH_VJgene,clonotype_IGL$IGL_VJgene,sep="|")

HLVJ <- unique(clonotype_IGL$HLVJ)

HLVJ <- V_J_gene_freq(HLVJ,"HLVJ","IGL")

HLVJ$comb <- HLVJ$HLVJ

HLVJ <- HLVJ %>% tidyr::separate(comb,c("IGHV","IGHJ","IGLV","IGLJ"),"[|]")

write.table(HLVJ,file="IGHL.VJ.txt",sep="\t",quote=F,col.names=T,row.names=F)

#IGHV-IGHJ-IGKV-IGKJ

clonotype_IGK$HKVJ <- paste(clonotype_IGK$IGH_VJgene,clonotype_IGK$IGK_VJgene,sep="|")

HKVJ <- unique(clonotype_IGK$HKVJ)

HKVJ <- V_J_gene_freq(HKVJ,"HKVJ","IGK")

HKVJ$comb <- HKVJ$HLVJ

HKVJ <- HKVJ %>% tidyr::separate(HKVJ,c("IGHV","IGHJ","IGKV","IGKJ"),"[|]")

write.table(HKVJ,file="IGHK.VJ.txt",sep="\t",quote=F,col.names=T,row.names=F)

##Fig2G

gene_freq <- function(datainput,genelist,type) {

##type should be IGHV、IGHJ、IGLV、IGLJ、IGKV、IGKJ

result_temp <- data.frame()

for (gene in genelist) {

data_temp <- datainput[datainput[[type]]==gene,]

freq_temp <- sum(data_temp$Frequency)

result1 <- data.frame(gene=gene,frequency=freq_temp)

result_temp <- rbind(result_temp,result1)

}

print(head(result_temp))

result_temp <- result_temp[order(result_temp$frequency,decreasing=T),]

return(result_temp)

}

## for IGH and IGL chain pairs

#HLVJ_top <- HLVJ_top %>% tidyr::separate(HLVJ,c("IGHV","IGHJ","IGLV","IGLJ"),"[|]")

head(HLVJ)

IGHV <- unique(HLVJ$IGHV)

IGHV_freq <- gene_freq(HLVJ,IGHV,"IGHV")

for (genetype in c("IGHV","IGHJ","IGLV","IGLJ")) {

assign(genetype,unique(HLVJ[[genetype]]))

assign(paste(genetype,"freq",sep="_"),gene_freq(HLVJ,get(genetype),genetype))

HLVJ[[genetype]] <- factor(HLVJ[[genetype]],levels=get(paste(genetype,"freq",sep="_"))$gene)

}

HLVJ$Clonotype <- paste("clonotype",seq(nrow(HLVJ)),sep="")

HLVJ <- HLVJ[order(HLVJ$Frequency,decreasing=T),]

HLVJ <- subset(HLVJ,select=c("IGHV","IGHJ","IGLV","IGLJ","Frequency","Clonotype"))

data_long <- to_lodes_form(data.frame(HLVJ),

key = "Type", value = "Group", id = "Cohort",

axes = 1:4)

p <- ggplot(data = data_long,aes(x = Type, stratum = Group, alluvium = Cohort, y = Frequency)) +

theme_bw() +

geom_alluvium() +

geom_flow(aes(fill = Clonotype,colour= Clonotype)) +

geom_stratum(show.legend=TRUE,width=0.4,aes(fill=Group)) +

ggfittext::geom_fit_text(stat = "stratum", aes(label = Group),width = 0.4, size=20,min.size = -6,grow=F) +

theme_minimal() +

theme(legend.position="none") +

theme(axis.text.x = element_text(color="black", size=25)) +

theme(axis.text.y = element_text(color="black", size=25)) +

xlab("") + ylab("")

pdf("IGHL.VJ.pdf",width=24,height=12)

p

dev.off()

## for chain IGH and IGL chain pairs

for (genetype in c("IGHV","IGHJ","IGKV","IGKJ")) {

assign(genetype,unique(HKVJ[[genetype]]))

assign(paste(genetype,"freq",sep="_"),gene_freq(HKVJ,get(genetype),genetype))

HKVJ[[genetype]] <- factor(HKVJ[[genetype]],levels=get(paste(genetype,"freq",sep="_"))$gene)

}

HKVJ$Clonotype <- paste("clonotype",seq(nrow(HKVJ)),sep="")

HKVJ <- HKVJ[order(HKVJ$Frequency,decreasing=T),]

HKVJ <- subset(HKVJ,select=c("IGHV","IGHJ","IGKV","IGKJ","Frequency","Clonotype"))

data_long <- to_lodes_form(data.frame(HKVJ),

key = "Type", value = "Group", id = "Cohort",

axes = 1:4)

p <- ggplot(data = data_long,aes(x = Type, stratum = Group, alluvium = Cohort, y = Frequency)) +

theme_bw() +

geom_alluvium() +

geom_flow(aes(fill = Clonotype,colour= Clonotype)) +

geom_stratum(show.legend=TRUE,width=0.4,aes(fill=Group)) +

ggfittext::geom_fit_text(stat = "stratum", aes(label = Group),width = 0.4, size=20,min.size = -6,grow=F) +

theme_minimal() +

theme(legend.position="none") +

theme(axis.text.x = element_text(color="black", size=25)) +

theme(axis.text.y = element_text(color="black", size=25)) +

xlab("") + ylab("")

pdf("IGHK.VJ.pdf",width=24,height=12)

p

dev.off()

##Fig2F. Isotype

isotypes <- c("IGHM","IGHD","IGHA1","IGHA2","IGHG1","IGHG2","IGHG","IGHG4","IGHE")

#result <- read.table("low-OPK-low-OPK-1.perCell.txt",header=T,sep="\t")

result <- result[result$c_gene_IGH != "None" & result$productive_IGH == "True",]

data <- result[result$c_gene_IGH %in% isotypes,]

data <- na.omit(data)

Isotype <- as.data.frame(table(data$c_gene_IGH))

colnames(Isotype) <- c("Isotype","Count")

Isotype <- Isotype[Isotype$Isotype != "None",]

cellnum <- sum(Isotype$Count)

Isotype$Frequency <- Isotype$Count/cellnum

Isotype <- Isotype[order(Isotype$Frequency,decreasing=T),]

Isotype$Isotype <- factor(Isotype$Isotype,levels=Isotype$Isotype)

write.table(Isotype,file="isotype.result.xls",sep="\t",quote=F,row.names=F)

## pie plot

num <- nrow(Isotype)

myPalette <- brewer.pal(num, "Set2")

Prop <- Isotype$Frequency

Prop_perc <- paste(round((100*Prop),2),"%",sep="") ## 0.0045 --> 0.45%

labels <- paste(Isotype$Isotype,paste("(",Prop_perc,")",sep=""),sep=" ")

pie_plot <- pie(Prop, labels=labels, border="white", col=myPalette)

pdf("isotype.freq.pieplot.pdf")

pie(Prop, labels=labels, border="white", col=myPalette)

dev.off()

if (file.exists("Rplots.pdf")) {

#Delete file if it exists

file.remove("Rplots.pdf")

}
